# Seasonal Spatial Distribution Characteristics and Patterns of the Squid *Uroteuthis duvauceli*, *Uroteuthis edulis*, *Loliolus sumatrensis*, and *Loliolus japonica* in the Southern Yellow and East China Seas: Predictions Under Different Climate Scenarios

**DOI:** 10.3390/ani15121744

**Published:** 2025-06-13

**Authors:** Min Xu, Hui Zhang, Bingqing Xu, Yong Liu, Linlin Yang

**Affiliations:** 1 Key Laboratory of East China Sea Fishery Resources Exploitation, Ministry of Agriculture and Rural Affairs, Shanghai 200090, China; xuminwzy@aliyun.com (M.X.); zhangh@ecsf.ac.cn (H.Z.); liuy@ecsf.ac.cn (Y.L.); 2 East China Sea Fisheries Research Institute, Chinese Academy of Fishery Sciences, Shanghai 200090, China; 3 Key Laboratory of Fisheries Remote Sensing, Ministry of Agriculture and Rural Affairs, Shanghai 200090, China; 4 Shandong Marine Resource and Environment Research Institute, Yantai 264006, China; xbq6688482@163.com

**Keywords:** population dynamics, stock assessment, climate warming, Kuroshio current, total allowable catch, fishery management, East China Sea Region, squid

## Abstract

For successful economic management of cephalopod fisheries, short-lived squid with a 1-year life span require stock assessment over a short timescale, accompanied by both in-season and real-time stock management. However, insufficient information is available about the dynamic distribution patterns of the squid *Uroteuthis duvauceli*, *Uroteuthis edulis*, *Loliolus sumatrensis*, and *Loliolus japonica* in China. Such information is vital for establishing a sustainable fisheries management system. In this study, seasonal fishery-independent bottom-trawling surveys were performed from 2018 to 2019 in the southern Yellow and East China Seas to assess the seasonal changes in the distribution of these species. Most *U. duvauceli* occurred at sea bottom temperatures (SBTs) of 24.52–26.96 °C and sea bottom salinity (SBS) of 30.2–31.54‰ during the summer and at 20.02–22.75 °C and 33.46–34.22‰ during the autumn. Most *U. edulis* occurred at an SBT of 18.29–19.61 °C and SBS 34.78–35.08‰ during the spring, at 18.63–19.43 °C and 34.43–34.66‰ during the summer, at 8.78–21.81 °C and 34.39–34.77‰ during the autumn, and at 17.90–21.55 °C and 34.34–34.61‰ during the winter. Overall, most *L. sumatrensis* occurred at 21.00–23.15 °C and 34.11–34.50‰ in autumn. *U. duvauceli* concentrated in the fishing grounds of Zhoushan during the spring, Lvsi and Zhoushan-Changjiangkou during the summer, Zhoushan and Yushan-Mindong during the autumn, and Wentai-Yushan during the winter. *L. sumatrensis* mainly occurred in the fishing grounds of Dasha, Changjiangkou-Zhoushan, and Mindong during the summer, Yushan during the autumn, and nearshore areas during the winter. Most *L. japonica* occurred in the fishing grounds of Haizhou Bay and Zhoushan-Yushan during the spring, Haizhou Bay during the summer, Lvsi during the autumn, and Haizhou Bay during the winter. Our results will be useful for determining the total allowable catch from squid fisheries in these areas in China.

## Simple Summary

Climate change is a vital factor impacting the distribution of benthic communities, including cephalopods. In this study, we identified the spatial seasonal distribution patterns of the economically important squid *Uroteuthis duvauceli*, *Uroteuthis edulis*, *Loliolus sumatrensis*, and *Loliolus japonica* under different climate scenarios (current, ssp1-2.6, ssp2-4.5, ssp3-7.0, and ssp5-8.5) in the southern Yellow and East China Seas. Furthermore, we identified the relationship between these species and environmental factors, including salinity and water temperature. We also predicted future variations in the annual habitat area range of each species under the different climate scenarios. Our results will be useful for understanding the population dynamics, making stock assessments, and developing forecasts for these species, thus supporting both fisheries and ocean management.

## 1. Introduction

Climate change is poised to affect coastal benthic communities, including cephalopods, via changes in water temperature [1,2]. As a result, these translate into greater impacts on ocean warming and higher risks of decreases in the number of species and populations, as well as local extinctions, along with functional and structural changes on an ecosystem scale [3,4,5]. Cephalopods, such as the squid *Uroteuthis duvauceli* (Teuthoidea, Loliginidae, Uroteuthis), *Uroteuthis edulis* (Teuthoidea, Loliginidae, Uroteuthis), *Loliolus sumatrensis* (Teuthoidea, Loliginidae, Lotiginidae), and *Loliolus japonica* (Teuthoidea, Loliginidae, Lotiginidae), have generally thrived with shifting coastal and ocean conditions [6]. In fact, some marine animal species either have been observed or are projected to shift poleward or into deeper waters under different climate change scenarios [7]. Furthermore, it is now obvious that the distribution of cephalopods is expanding latitudinally [8].

Cephalopods, including squid, are important trophic mid-points on the food web. They are opportunistic predators that prey on numerous species and, in turn, are important prey for marine mammals and seabirds [9]. Furthermore, they have great phenotypic plasticity, enabling them to rapidly adapt to changing environments [10]. This has led to increasing numbers of squid, particularly when their predators and competitors have been overfished [10].

In previous studies, we found that *Euprymna morsei* would experience larger negative impacts from climate changes compared with *Euprymna berryi* [11]. The habitat of *Sepiella maindroni* would shift to the south first and then to the north of the East China Sea, and the habitat area of *Sepia kobiensis* would increase with rising CO_2_ emissions [12]. The central distribution of *Sepia esculenta* occurs at a latitude of 28.00° N in autumn and winter [13]. The annual mean habitat area of *Amphioctopus fangsiao* would shrink significantly by both 2050 and 2100, and the annual mean habitat of *Octopus variabilis* will shift northward offshore by 2050 and 2100 [14]. The most beneficial case for *Abralia multihamata* in terms of average habitat area occurs under SSP3-7.0 in 2050 [15]. *Amphioctopus ovulum* can be expected to expand to the northeast and southwest independently under the most likely global warming scenarios [16]. High-value areas of *Loliolus beka* and *Loliolus uyii* include inshore areas of the southern Yellow and mid-East China Seas during the autumn [17].

*U. duvauceli* is a warm species found in waters of the Indian–West Pacific region, where it is a major component of artisanal squid fisheries [18]. In addition, it is caught via trawl fishing in India, with most of the catch being used for frozen and value-added products destined for Europe [18]. It is also found off the Chinese coast, including the South China Sea, Taiwan Strait, and southern East China Sea [19]. It is a common bycatch species of trawling, elver nets, light-liftnet, and torch-light fisheries in China [20]. *U. duvauceli* sits at a trophic level of 2.85 and has a ~99-day life span, during which it mainly feeds on fish, crustaceans, other cephalopods, chaetognaths, nematodes, polychaetes, molluscs, and diatoms [19]. Parent cohorts occur from May to June and from July to September. The annual catch of *U. duvauceli* in China was reported to be 15,000 to 20,000 tons [19].

*U. edulis* occurs in spring, summer, and autumn cohorts in March–May, June–July, and August–October, respectively [21]. It is mainly distributed on the continual shelf of the East China Sea (30.5° N to the south) and at a depth of ~60–200 m [22]. The main fishing grounds for this species occur at 28–30.5° N 124–126.5° E and 27–28.5° N 122.5–125.5° E [23]. The main fishing period is May–October, peaking in June–September [24]. It is fished using bottom pair trawlers and bottom single trawlers (June–August), and so-called ‘squid jigging’ by using light, light-liftnets, and light purse seine (March–November) [24]. In Taiwan, the use of torchlight, trawler, and pole-and-line boats also occurs [25]. In China, the annual catch was estimated to be 15,000 tons [26].

*L. sumatrensis* is mainly distributed in the South China Sea, whereas *L. japonica* occurs mainly in the Bohai and Yellow Seas [27]. Both have a mantle length of 8–11 cm and a ~1-year lifespan [28]. The mean landing of *L. japonica* was 50,000 tons annually from 1970 to 1979, fluctuating between 13,000 and 108,000 tons and over a period of 6–8 years, with strong generations in 1964 to 1967 and 1972 to 1975, but weak generations in 1960 to 1963, 1968 to 1971, and 1976 to 1978 [27].

Finally, the dominant squid species in the East China Sea region has shifted from *Sepiella maindroni* and *Sepiella japonica* to *U. duvauceli* and *U. edulis*. However, the abundance of *U. duvauceli* is currently decreasing, while that of *U. edulis* is increasing, indicating a potential case of species succession in this area [29]. Thus, understanding the distribution of these squid species across the seasons and how this might be impacted by climate change will be important to setting and managing relevant fisheries in this region.

Therefore, we investigated the seasonal spatial distribution characteristics and patterns of *U. duvauceli*, *U. edulis*, *L. sumatrensis*, and *L. japonica* in the East China Sea region, and their correlation with environmental variables, such as water temperature and salinity. Furthermore, we modeled how their annual habitat area range might differ under different climate scenarios, and across different seasons. Such information about the distribution patterns, migration routes, and biologies of these species will contribute to stock assessments, understanding and predicting their population dynamics, developing forecasts, and, thus, supporting fisheries management in the future.

## 2. Materials and Methods

### 2.1. Geographical Information, Survey and Sampling Procedures

The Yellow Sea is located north of Shanghai, China and is enclosed by the Bohai Sea and mainland Chinese coast in the northwest, which separates it from the Sea of Japan and the northwest Pacific Ocean to the east. The East China Sea is influenced by the warm Taiwan and Kuroshio currents, characterized by high water temperatures and high salinity, and by coastal currents in the northern East China Sea, with low water temperatures and salinity. Such differences result in complex oceanographic conditions in the southern East China Sea. Northeastern Taiwan is located between the continental shelf of the East China Sea and the Okinawa Trench, and is affected by the southwest monsoon in summer, which mixes the northward Taiwan warm current, the southward mainland coastal cold current, and the Kuroshio current to form frontal sea areas. From June to September, the Taiwan Strait Warm Current is pushed forward by a southwest monsoon. After October, a northeast monsoon leads to inflow of the Mainland China Coastal Current. This area is also affected by the northeast monsoon in winter, which transports cold waters with low salinity from the mainland coasts to the area. Furthermore, in this area, there is a year-round upwelling around Peng-Chia Island [30], and several typhoons bring particulate organic carbon flux from June to September [31]. Thus, this area has an upwelling current and fronts in the inshore and offshore areas, respectively.

**Figure 1:**
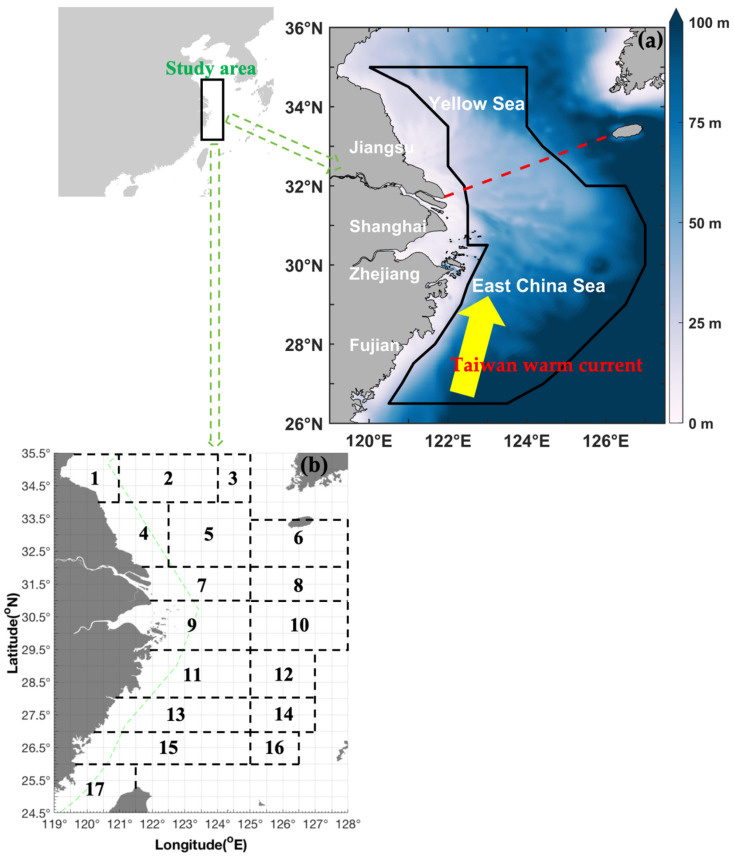
Survey map information. (**a**) Map of the study area in the East China Sea region (26.50° N–35.00° N 120.00° E–127.00° E), denoted by a black solid line. The area includes the southern Yellow and East China Seas adjacent to the coastlines of Fujian, Zhejiang, Shanghai, and Jiangsu. The color bar denotes the depth range (0 m–100 m). The red-dashed line indicates the boundary between the Yellow Sea and East China Sea; the yellow arrow indicates the Taiwan warm current. (**b**) Fishing grounds: (1) Haizhou Bay, (2) Lianqingshi, (3) Liandong, (4) Lvsi, (5) Dasha, (6) Shawai, (7) Yangtze River mouth, (8) Jiangwai, (9) Zhoushan, (10) Zhouwai, (11) Yushan, (12) Yuwai, (13) Wentai, (14) Wenwai, (15) Mindong, (16) Minwai, and (17) Minzhong.

We performed scientific fishery-independent bottom-trawling surveys in 2018 and 2019 in the southern Yellow and East China Seas. The surveys used a trawl net with a cod-end mesh size of 20 mm towed by fisheries research vessels during spring (April–May 2019), summer (August–September 2019), autumn (November 2018), and winter (January 2019). The sample trawls were conducted in a grid characterized by latitude and longitude spacings of 30 min × 30 min. Each tow was assigned to a specific grid cell according to the survey location (Figure 1). The average trawl speed was 3 knots. All tows were conducted for 1 h at each station. The motor-trawl prohibition line indicates the boundary of areas in which fishing is forbidden to prevent the destruction of aquatic resources by wheel trawling.

Environmental conditions, such as sea temperature and salinity, are known to impact squid abundance by directly and indirectly impacting on biological processes [18]. Furthermore, loliginid squid have a strong preference for a benthic environment for feeding and spawning [32]. Thus, environmental factors were measured at each station using a profiler (SeaBird-Scientific, Bellevue, WA, USA; SBE-19). Sea surface salinity (SSS) and sea surface temperature (SST) were measured at 3 m below the surface, whereas sea bottom salinity (SBS) and sea bottom temperature (SBT) were measured 2 m above the sea bottom at depths < 50 m and at 2–4 m above the bottom at depths >50 m. The measured depth data were impacted by variation in the tidal range.

The samples from each survey station were transported to the laboratory for species identification. In addition, the catch per unit effort (CPUE) was determined in terms of seasonal total biomass density (CPUE_n_; ind·h^−1^) and seasonal total biomass weight was recorded to the nearest 0.10 g of wet weight for the evaluation of the catch per unit effort (CPUE_w_; g·h^−1^) (Table 1). The average individual weight (AIW) was defined as the CPUE_w_ divided by the CPUE_n_ at each station.

### 2.2. Ensemble Model, Selection of Environmental Variables, and Evaluations

Species distribution models (SDMs) are extensively applied to identify the habitat distribution variations of species [33]. We used ten algorithms to forecast the habitat distribution of *U. duvauceli*, *U. edulis*, *L. sumatrensis*, and *L. japonica*: extreme gradient boosting training (XGBOOST), surface range envelope (SRE), random forest (RF), multiple adaptive regression splines (MARS), generalized linear models (GLMs), generalized boosting models (GBMs), generalized additive models (GAMs), flexible discriminant analysis (FDA), classification tree analysis (CTA), and artificial neural networks (ANNs).

We used the ‘biomod2’ package in the ensemble SDM platform. To run the model, the data set was separated into categories of 1 (presence) and 0 (absence), and a 20%: 80% split was then randomly applied for testing and training data independently to construct the ten algorithms using the random cross-validation method [34]. We used the mean survey data over 4 months (November, January, May, and August) to produce the annual model, and used different seasonal data to produce the seasonal models. The performance of each algorithm was assessed by the index of the area under the true skill statistic (TSS) and the receiver operating characteristic curve (ROC) [35]. We selected those algorithms that performed best (AUC > 0.8) and combined them into an ensemble model using the weighted average method (details in Supplementary File S2). Future climate data were obtained from the Coupled Model Intercomparison Project Phase 6 (CMIP6), and environmental data, such as SSS, SBS, SST, and SBT, were obtained from the website Bio-ORACLE (https://bio-oracle.org/index.php, accessed on 22 April 2025). The four shared socioeconomic pathway (SSP) scenarios (SSP1–2.6, SSP2–4.5, SSP3–7.0, and SSP5–8.5) for 2040–2050 (the 2050s) and 2090–2100 (the 2090s) [36] were used in this study.

Although foundational, climate models have intrinsic limitations that can introduce biases in projected environmental factors. Thus, bias correction of climate model raw data is essential to enhance the credibility of habitat distributions under future climate scenarios [37]. The delta method is a prevalent technique in fisheries habitat prediction that effectively mitigates such biases [38,39,40]. We used this approach to calculate climate differences between contemporary and future data sets by applying corrections to raw data [41].

## 3. Results and Discussion

### 3.1. Seasonal Spatial Variation and Migration of U. duvauceli

**Figure 2:**
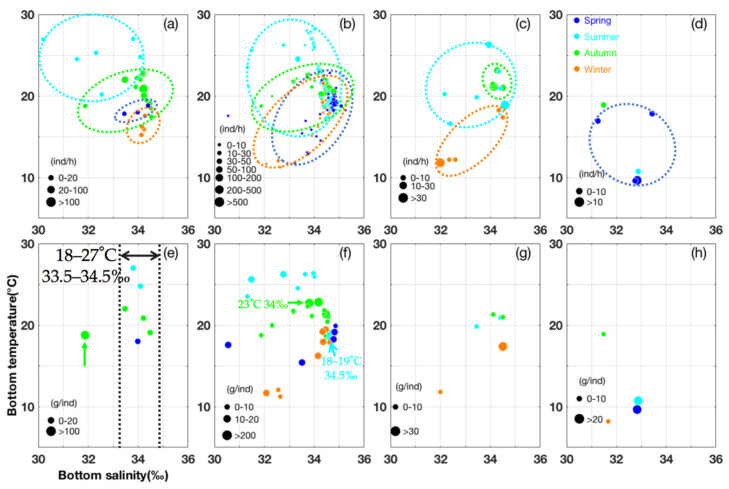
Correlation between sea bottom salinity (‰) and sea bottom temperature (°C) for CPUE_n_ (**a**–**d**) and AIW (**e**–**h**) for (**a**,**e**) *Uroteuthis duvauceli*, (**b**,**f**) *Uroteuthis edulis*, (**c**,**g**) *Loliolus sumatrensis*, and (**d**,**h**) *Loliolus japonica*. The spring, summer, autumn, and winter data are indicated by blue, light blue, green, and brown circles, respectively.

In India, most *U. duvauceli* were found at a depth of ~50–60 m, occurring up to a depth of 120 m [42]. In our study, the depth ranges were similar, comparing spring with summer and autumn with winter, indicating the movement to deeper areas after the autumn (Table 2). The seasonal order of SST was summer > autumn > spring > winter, with a range of 15–29 °C (Table 2). SST and SBT ranges were similar in spring, autumn, and winter (Table 2). SSS and SBS ranges were similar in autumn and winter, whereas SBS was higher than that of SSS in spring and summer (Table 2). In addition, in summer, more *U. duvauceli* were found at an SBT of between 24.52 °C and 26.96 °C and an SBS of 30.2‰ and 31.54‰ (Figure 2). In autumn, more *U. duvauceli* occurred at an SBT of 20.02 °C and 22.75 °C and an SBS of between 33.46‰ and 34.22‰, with larger individuals surviving at an SBT of 18.75 °C and SBS of 31.88‰ (Figure 2).

In China, Zhang et al. (2010) identified the spawning period of *U. duvauceli* as June–September; the highest seasonal biomass occurred in the order autumn > summer > winter > spring; *U. duvauceli* underwent seasonal reproduction–feeding migration over a short distance from north to south and from deeper to shallower areas in the southern East China Sea [19]. In India, mature *U. duvauceli* undergo migrations to inshore spawning grounds [43] for congregation and egg laying; spawning aggregations occurred annually in nearshore spawning grounds during September–October, and the peak abundance occurred while temperatures were increasing in near-shore waters, coinciding with the retreating monsoons in September and during the transition from winter to summer [44]. In this study, the mean and upper limit values of CPUE_w_, CPUE_n_, and AIW were all in the order autumn > summer > winter > spring (Table 3).

**Figure 3:**
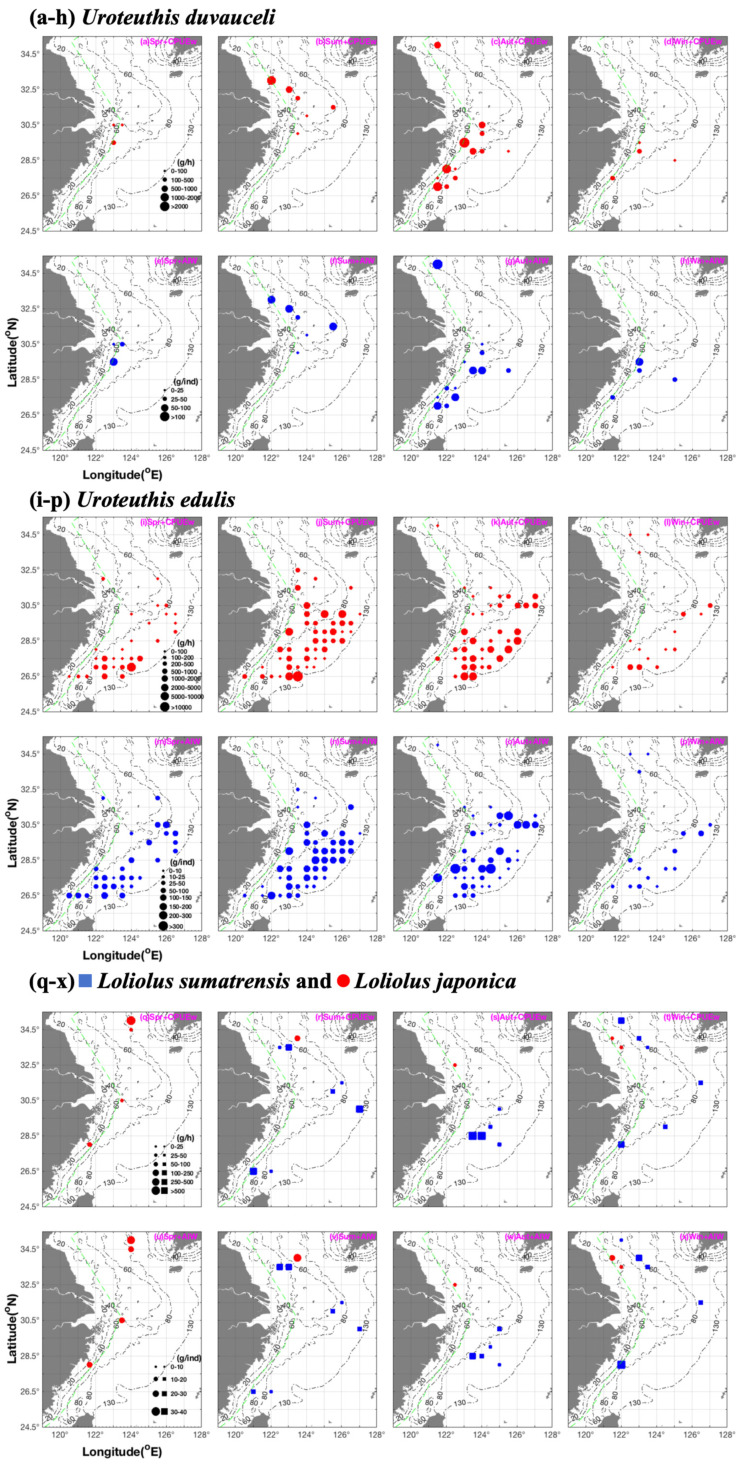
Seasonal distribution patterns of CPUE_w_ (g·h^−1^) and AIW (g·ind^−1^) for *Uroteuthis duvauceli* (**a**–**h**) and *Uroteuthis edulis* (**i**–**p**), and CPUE_w_ (g·h^−1^) and AIW (g·ind^−1^) of *Loliolus sumatrensis* (blue squares) and *Loliolus japonica* (red circles) (**q**–**x**). The green dashed line indicates the motor-trawl prohibition lines. A black dash-dot line represents the depth gradient (20–130 m).

In China, most *U. duvauceli* biomass occurred at 26.75–27.75° N 123.25–124.25° E with an SBT of 17–27 °C [12]. *U. duvauceli* made a short migration from the Wentai fishing grounds in spring and autumn to the fishing grounds of Mindong and Minwai-Wenwai in summer and winter, at a depth of 90–100 m [19]. The Taiwan strait and East Sea continental shelf areas were spawning and nursery grounds [20]. In this study, *U. duvauceli* were found to be concentrated in the Zhoushan fishing ground in spring, the fishing grounds of the Lvsi and the Zhoushan-Yangtze River mouth in summer, the fishing grounds of Zhoushan and Yushan-Mindong in autumn, and the fishing grounds of Wentai-Yushan in winter (Figure 3). In summer, the mean CPUE_w_ and AIW in Lvsi were higher than that in the Zhoushan-Yangtze River mouth (Figure 3). In autumn, more juveniles and fewer larger individuals were found in the inshore and offshore areas of the Yushan-Mindong fishing grounds (Figure 3). 

### 3.2. Seasonal Spatial Variation and Migration of U. edulis

During the 1990s, the increase in catches in the East China Sea appeared to be caused by a favorable warm environment, and positive relationships have been reported between catches of *U. edulis* and SST across seasons in the East China Sea [45]. In this study, in the East China Sea region, *U. edulis* occurred at a depth of 10–140 m in areas where SST was higher than the SBT in spring, autumn, and winter. Paralarval and juvenile *U. edulis* were found in different seasons, with a minimum sex maturity mantle length of 67 mm [22]. Sex maturity was determined by a mantle length of males and females of >120 mm and >140 mm, respectively, with a relationship of W = 2.559 × 10^−3^ × L^2.185^ between mantle length (L; unit: mm) and weight (W; unit: g) [23]. In this study, most *U. edulis* were found in an SBT of 18.29–19.61 °C and SBS of 34.78–35.08‰, with juveniles (estimated mantle length = 60 mm) occurring in an SBT of 15.4–19.88 °C and SBS of 33.51–34.86‰ during spring (Figure 2). The spring cohort occurred in temperatures ranging from 19.34 °C to 23.91 °C, with a hatching water temperature of 18.5 °C [46]. SST and SSS in spring were estimated to be 22–24 °C and 34–36‰ respectively [47]. Juveniles (estimated mantle length of ~60 mm) occurred in an SBT of 17.95–26.33 °C and SBS of 31.31–34.68‰, with most found in an SBT of 18.63–19.43 °C and SBS of 34.43–34.66‰ during the summer (Figure 2). The summer cohort occurred at 15.91–19.43 °C, with a hatching water temperature of 21 °C [46]. SST and SSS in summer were estimated to be 23–24 °C and 31–34‰, respectively [47]. Juveniles (estimated mantle length of ~45 mm) occurred in an SBT of 18.26–22.28 °C and SBS of 31.88–34.71‰ (Figure 2). In autumn, adults (estimated mantle length >173.5 mm) occurred in an SBT of 19.08–22.83 °C and SBS of 33.8–34.57‰, with most found in an SBT of 18.78–21.81 °C and SBS of 34.39–34.77‰ (Figure 2). In winter, most adults occurred in an SBT of 17.9–21.55 °C and SBS of 34.34–34.61‰, whereas most juveniles (estimated mantle length < 32 mm) occurred at 11.24–12.07 °C and SBS of 32.56–32.65‰ (Figure 2).

In terms of seasonal spatial distribution pattern, the highest biomass occurred in the order summer > spring (and autumn) > winter (Table 3), which is consistent with previous results [23], indicating that *U. edulis* has a specific preference for higher water temperatures. The mean value and upper limit values of CPUE_n_ occurred in the order autumn (and summer) > spring > winter (Table 3). Maximum mean mantle length and dominant mantle length group were also found in autumn [22]. In this study, the mean and upper limit values of AIW occurred in the autumn > summer > spring > winter (Table 3).

In terms of seasonal spatial distribution patterns, the highest biomass in spring was concentrated in the southern Wentai fishing grounds (Figure 3). By contrast, larger individuals and numerous juveniles occurred in the mid-Mindong fishing grounds (Figure 3), suggesting the existence of spawning ground in the southern East China Sea. Most of the biomass was concentrated in the southern East China Sea, with the highest abundance occurring along the longitudinal line of 124° E (Figure 3). Liao et al. (2018) reported a high abundance at 27–28° N during spring [48]. For the summer, our results indicate a potential spawning ground off Zhoushan island; by contrast, in the northern East China Sea, juvenile *U. edulis* migrated northward (Figure 3). In autumn, most *U. edulis* occurred along a longitudinal line of 123° E to 123.5° E and 125° E to 126° E (Figure 3). In autumn and winter, juveniles and larger individuals occurred in inshore and offshore areas, respectively (Figure 3).

In terms of their migration route, previous studies have identified paralarval *U. edulis* in inshore waters off northern Taiwan island (indicating nursing grounds) in January to February, and spawning grounds in upwelling water areas of Peng-Chia (~18.5 °C) after March [25]. From January to March, the northeast monsoon prevails around northeastern Taiwan, causing the Kuroshio reflux to move southwestward under the influence of the continental coastal currents, which supports the spring cohort paralarvae in their movement with this current, causing their dispersal throughout the coastal nursery areas around northern Taiwan [49]. With the increasing water temperature and continuous strengthening warm currents in spring, the parent cohort moves northwestward from offshore overwintering grounds in southeastern Zhejiang. The recruitment cohort migrates to areas at 26–29° N at a depth of 100–200 m from April to May, which is likely attributed to continuous strengthening of warm currents and southwest monsoon, after which they migrate northwestward to the continental area of 27–28° N 122.5–125.5° E and 28–30.5° N 124–126.5° E at a depth of 60–100 m from June to August [23]. After October, a strengthening northeast monsoon and decreasing offshore water temperature forces *U. edulis* to retreat to a warmer offshore environment in the southern East China Sea for overwintering [25]. In addition, the summer cohort (at 21 °C) is spawned in Zhoushan at 29–31.5° N 122–125° E, and juveniles migrate to the mid-East China Sea under the influence of Changjiang diluted water (CDW), and then to the northeastern East China Sea with the Taiwan warm current [46].

### 3.3. Seasonal Spatial Variation and Characteristics of L. sumatrensis and L. japonica

For *L. sumatrensis*, the depth range was similar in summer, autumn, and winter (Table 2). SST and SBT ranges were similar in autumn and winter (Table 2). SSS values showed that the cohort was influenced by CDW in summer, and distributed in offshore areas with an SSS of 32–34.5‰ in autumn and winter (Table 2); juveniles (<10 g∙ind^−1^) occurred at an SBT of 19.82–20.91 °C and SBS of 33.45–34.4‰ (Figure 2). In autumn, most *L. sumatrensis* were found at an SBT of 21–23.15 °C and SBS of 34.11–34.5‰, and juveniles occurred at an SBT of 21–21.31 °C and SBS of 34.11–34.5‰ (Figure 2). In winter, most juveniles occurred at an SBT of 11.78 °C and SBS of 32‰ (Figure 2). The mean CPUE_w_ and CPUE_n_ values occurred in the order of autumn > summer (and winter) (Table 3). The mean AIW value in winter was higher than that in the autumn (Table 3). In addition, in terms of seasonal spatial distribution pattern, in summer, *L. sumatrensis* occurred in the Dasha, Changjiangkou-Zhoushan, and Mindong fishing grounds, concentrated in inshore areas for feeding and as nursery sites (Figure 3). In autumn, the species was concentrated in the Yushan fishing ground with a high juvenile biomass, suggesting this is as a spawning ground (Figure 3). In winter, *L. sumatrensis* was scattered among nearshore areas, which also acted as nurseries for this species (Figure 3).

The depth range of *L. japonica* was 15–83 m (Table 2). This species is thought to prefer colder waters because they were found at 8 °C in winter, 10–11 °C in summer, and 10–18 °C in spring (Table 2). SSS and SBS value ranges were similar in autumn and winter, whereas SBS was higher than SSS in spring and summer (Table 2). The mean and upper limit values of CPUE_w_ were highest in spring; the seasonal order of AIW was summer > spring > winter > autumn (Table 3). *L. japonica* was found in Haizhou Bay and Zhoushan-Yushan in spring, Haizhou Bay in summer, Lvsi in autumn, and Haizhou Bay fishing grounds in winter (Figure 3). Du et al. (2017) reported that the southernmost distribution of the species was at the Zhoushan-Yushan fishing grounds, and that their spatial distribution patterns were affected by seasonal variations [50]. Wei (1966) has suggested *L. japonica* makes a spawning migration after March, with one cohort moving to the coastal areas of northern Jiangsu, whereas juvenile nursery and feeding grounds occur in Haizhou Bay in May, with migration to the middle Yellow Sea at 34–37° N 122–124° E in September–October for overwintering [51].

### 3.4. Model Evaluation and Suitable Environmental Factors and Habitat

In this study, the RF algorithm performed the best for each of the four squid species (Figures S1–S8).

Rodhouse (2014) has argued that, in squid populations, recruitment variability is driven by the environment, presenting a challenge to the management of squid fisheries [52]. The environmental sensitivity of squids is correlated with multiple drivers influencing their distribution, abundance [53], and migration to favored environments [54] or to areas that maximize their spawning success [55]. Water temperature is a main factor affecting the distribution pattern of squid [56] and, therefore, changes in water temperature could explain the observed increases and potential expansion of populations. The influence of temperature has been related to the variability in squid abundance, availability of spawning grounds [57], strength of spawning activity [58], and onset of migrations [59]. Squid might select an optimum temperature range to spawn by undertaking spawning migrations in an attempt to maximize their hatching success [55]. Wang et al. (2015) examined the SST of squid fishing grounds and found markedly higher SST values during the years when the catch ratios were high [20].

**Figure 4:**
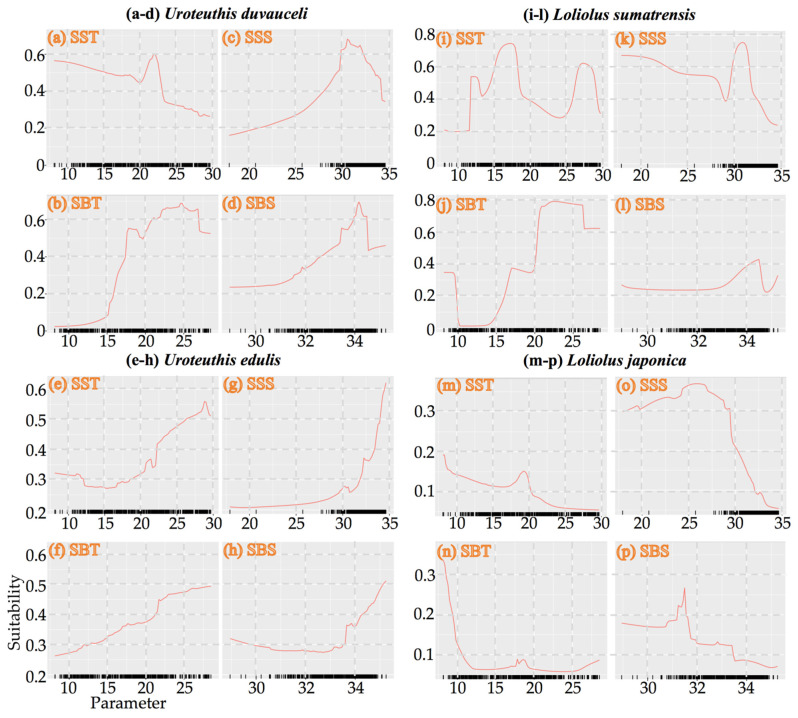
Suitability responses to environmental variables: SST (10–30 °C), SSS (20–35‰), SBT (10–25 °C), and SBS (30–34‰): (**a**–**d**) *Uroteuthis duvauceli*; (**e**–**h**) *Uroteuthis edulis*; (**i**–**l**) *Loliolus sumatrensis*; (**m**–**p**) *Loliolus japonica*.

**Figure 5:**
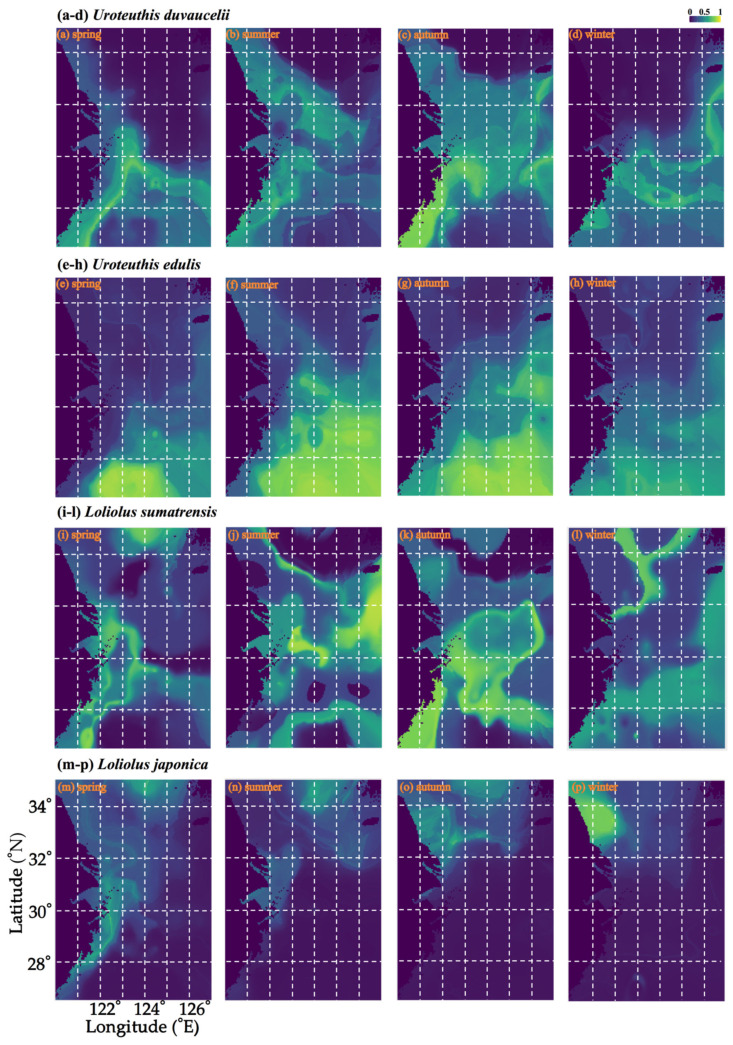
Seasonal spatial distribution patterns in the study area as predicted by the ensemble model across seasons: (**a**–**d**) *Uroteuthis duvauceli*; (**e**–**h**) *Uroteuthis edulis*; (**i**–**l**) *Loliolus sumatrensis*; (**m**–**p**) *Loliolus japonica*. The blue–green color range indicates low-to-high suitability.

For *U. duvauceli*, variability in SBT was most important in ANN, CTA, GAM, GBM, GLM, MARS, RF, SRE, XGBOOST, and ensemble model algorithms, whereas variability in SBS was most important in the FDA algorithm (Figure S9). The suitability index increased with increasing SBT, SSS, and SBS, but decreased with increasing SST (Figure 4). In spring, *U. duvauceli* concentrated in the vicinity of the motor-trawl prohibition lines in the East China Sea, off the Zhoushan islands, and in waters outside the Yangtze estuary; in summer, some of the population moved to the Lvsi fishing ground, and became concentrated in fishing grounds around the Yangtze River estuary; in autumn and winter, *U. duvauceli* retreated to the coasts of the East China Sea (Figure 5).

For *U. edulis*, variability in SST was most important in the ANN and GBM algorithms; variability in SBT was most important in the SRE algorithm; variability in SSS was most important in the FDA, GAM, GLM, MARS, RF, XGBOOST, and ensemble model algorithms; and SBS was most important in the CTA algorithm (Figure S10). The suitability index increased with the environmental factors SST, SBT, SSS, and SBS (Figure 4). Suitable SST, SBT, SSS, and SBS were 28–29 °C, 18–21 °C, 32–34‰, and 34–34.5‰, respectively (Figure 4). Numerous *U. edulis* were caught in an SBT of 16–19 °C and SBS 33.5–34.6‰ of Taiwan island [46]. In spring, the species concentrated on the southern East China Sea at 27–27.5° N 121.5–125.5° E, while, in summer, they concentrated in offshore areas at 26.5–30° N and in areas >80 m in depth in the East China Sea in autumn and winter. It appears that suitable habitats in summer and autumn were larger and expanded northwards toward the coastline, whereas these retreated southwards to areas near the edge of the East China Sea continental shelf in spring and winter (Figure 5).

For *L. sumatrensis*, variability in SST was most important in the FDA and GAM algorithms; variability in SBT was most important in the ANN, CTA, MARS, RF, XGBOOST, and ensemble model algorithms;; and variability in SBS was most important in the GBM, GLM, and SRE algorithms (Figure S11). *L. sumatrensis* concentrated in waters around the Yangtze River estuary in summer, and moved to coastal and offshore areas of the East China Sea in autumn, before scattering across the Yellow and East China Seas in winter (Figure 5).

For *L. japonica*, variability in SSS was most important in the GAM, GLM, and ensemble model algorithms, variability in SBS was most important in the CTA, GBM, RF, SRE, and XGBOOST algorithms, whereas variability in SBT was most important in the ANN, FDA, and MARS algorithms (Figure S12). The suitability index decreased with increasing SST, SBT, SSS, and SBS (Figure 4). *L. japonica* concentrated in coastal areas of the East China Sea in spring, Liandong fishing grounds in summer, and migrated to the southern Yellow Sea in autumn and winter (Figure 5).

### 3.5. Habitat Predictions Under Different Climate Projections

Aquatic ecosystems are projected to experience distribution shifts under future climate change scenarios, which might affect recruitment success and survival of the early stages of aquatic organisms [60]. Jackson (2004) argued that tropical species appear to tolerate and even thrive in very warm conditions and, thus, that these species are likely to be favored by increases in water temperature in response to climate change [56]. The East China Sea region, including the southern Yellow and East China Seas, experienced continuous rapid warming, with SST increasing by ~0.98 ± 0.19 °C from 1958 to 2018 [61].

**Figure 6:**
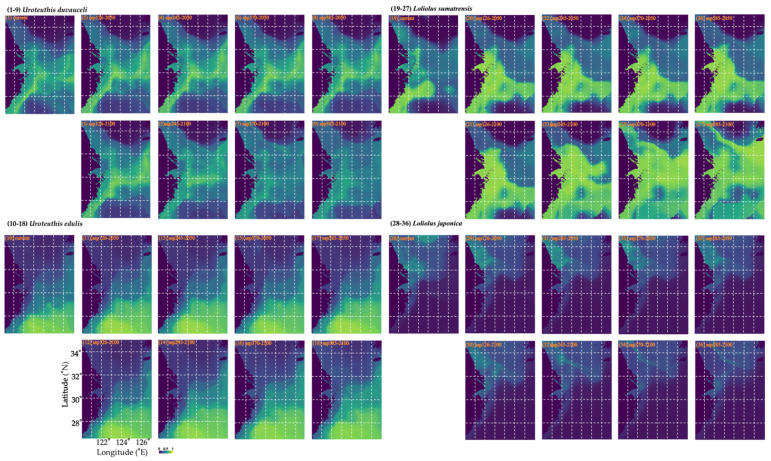
Predicted spatial habitat distribution patterns of *Uroteuthis duvauceli*, *Uroteuthis edulis*, *Loliolus sumatrensis*, and *Loliolus japonica* in terms of (1, 10, 19, 28) annual mean habitat; (2, 11, 20, 29) under SSP1-2.6 in 2050; (3, 12, 21, 30) under SSP1-2.6 in 2100; (4, 13, 22, 31) under SSP2-4.5 in 2050; (5, 14, 23, 32) under SSP2-4.5 in 2100; (6, 15, 24, 33) under SSP3-7.0 in 2050; (7, 16, 25, 34) under SSP3-7.0 in 2100; (8, 17, 26, 35) under SSP5-8.5 in 2050; and (9, 18, 27, 36) under SSP5-8.5 in 2100. The blue–green bar indicates the range from low to high suitability.

We examined projected changes in the habitat distribution of the four squid species in response to different climate change scenarios. We found that *U. duvauceli* would experience a loss of >50% under SSP370-2100 and SSP585-2100, compared with a 10–30% loss under other scenarios (Table 4 and Figure 6). By contrast, most percentage gains ranged from 10% to 20% (Table 4). For *U. edulis*, the percentage gain was 20–30% under all scenarios and this species would become concentrated mainly in the central and southern East China Sea (Table 4 and Figure 6).

*L. sumatrensis* would be concentrated in the vicinity of the motor-trawl prohibition lines and the northern East China Sea, benefitting from warming occurring under different climate scenarios (Table 4 and Figure 6). This species would become concentrated around coastal areas in the East China Sea under the current scenario, expanding to the whole East China Sea under SSP126-2050 and SSP126-2100, whereas they would transfer to the southern Yellow Sea under SSP245-2050, SSP245-2100, SSP370-2050, SSP370-2100, SSP585-2050, and SSP585-2100 (Figure 6).

By contrast, *L. japonica* would become concentrated in the middle Yellow Sea and Lvsi fishing grounds under the current scenario, changing to coastal areas of the southern Yellow Sea under SSP126-2050 and expanding to Yangtze river water areas and offshore areas in the southern Yellow Sea under SSP126-2100, to the East China Sea under SSP245-2050, SSP370-2050, and SSP585-2050, and to the Yellow and East China Seas under SSP245-2100, SSP370-2100, and SSP585-2100, indicating that they would be negatively impacted by warming seas under these scenarios (Table 4 and Figure 6).

The suitable habitat range area across the seasons and climate scenarios of four species is detailed in Figures S13–S20.

Water temperature tolerance and prey availability are also important in determining variations in suitable habitat areas. Payne et al. (2016) have argued that water temperatures for the performance in wild aquatic animals were strongly correlated with warm boundary temperatures, and that decreasing environmental heating tolerance mirrored the change in physiological heating tolerance [62]. Liu et al. (2023) have found that the predicted results from using a prey–predator species distribution model for a piscivorous fish (Japanese Spanish mackerel) showed higher precision when considering prey distribution, such as that of Japanese anchovy [36].

### 3.6. Fishery Management Suggestions

Currently, there is insufficient information about the seasonal spatial distribution patterns and population dynamic variations of *U. duvauceli*, *U. edulis*, *L. sumatrensis*, and *L. japonica* to establish a sustainable fisheries management system. These short-lived squids require assessment and management over a shorter timescale, often necessitating in-season assessment and real-time management of population and cohorts [63]. Their resource specific management is limited to a minimum legal size at harvest and seasonal fishery closures. The most common means of managing cephalopod fisheries is by regulating fishing effort, which reduces the risk of recruitment and parent overfishing [64]. Although squid are capable of recovering from low biomass levels, stock depletion is likely when heavy fishing pressure coincides with unfavorable environmental conditions. Thus, it is necessary to prioritize the protection of spawning and nursery grounds, adjusting fishing quotas based on seasonal distribution shifts, long-term monitoring of environmental variables, and the abundance and survival rates of recruitment and parent cohorts to enable the development of successful squid fisheries in response to climate change. In future, it will be necessary to perform comparative surveys to determine the survey grid spacing and the mapping of stations to correct and balance the bias from the surveys.

## 4. Conclusions

Further research is needed for a better understanding of the changes in distribution and migration patterns of *U. duvauceli*, *U. edulis*, *L. sumatrensis*, and *L. japonica*, and in their reproduction–recruitment processes under different environmental regimes, integrating social, economic, and ecological considerations. Our study indicates how to better assess the underlying causes of the sudden changes in abundance, and to further understand the life history of these species. This is an initial step in the development of predictive tools useful for fishery resource management in this region.

## Figures and Tables

**Table 1:** Seasonal total biomass density (CPUE_w_) and density (CPUE_n_) of *Uroteuthis duvauceli*, *Uroteuthis edulis*, *Loliolus sumatrensis*, and *Loliolus japonica* in the study area.

Season	*U. duvauceli*		*U. edulis*		*L. sumatrensis*		*L. japonica*	
	CPUE_w_	CPUE_n_	CPUE_w_	CPUE_n_	CPUE_w_	CPUE_n_	CPUE_w_	CPUE_n_
Spring	205.54	9	12,201.04	494	/	/	459.16	19
Summer	3202.84	52	47,658.02	1041	1137.32	92	26	2
Autumn	9495.05	525	29,493.32	2532	1543.66	114	2.688	5
Winter	590.2	14	2606.87	145	701.9	53	28.3	3
Total	13,493.63	600	91,959.25	4212	3382.88	259	516.148	29

**Table 2:** Seasonal in situ ranges of environmental variables for each squid species in the study area ^a^.

Variable	Spring	Summer	Autumn	Winter
*Uroteuthis duvauceli*				
Depth (m)	49–61	19–68	36–97	46–105
SST (°C)	17.17–18.58	25.97–29.09	18.66–23.13	15.03–17.54
SBT (°C)	17.83–18.85	20.14–26.97	17.45–22.83	15.24–17.57
SSS (‰)	30.46–32.02	29.81–32.82	31.86–34.38	34.06–34.18
SBS (‰)	33.45–34.4	30.2–34.08	31.88–34.53	34.12–34.27
*Uroteuthis edulis*				
Depth (m)	22–140	10–133	35–135	40–126
SST (°C)	13.28–25.99	25.25–29.5	18.66–26.29	11.21–22.34
SBT (°C)	11.73–22.79	17.23–28.19	17.88–23.15	11.24–21.55
SSS (‰)	30.08–34.63	27.69–34.3	31.86–34.45	32.16–34.52
SBS (‰)	30.55–35.25	31.31–34.68	31.88–35.07	32.08–34.61
*Loliolus sumatrensis*				
Depth (m)	/	31–97	54–100	49–90
SST (°C)	/	25.11–29.67	21.46–23.66	11.75–18.06
SBT (°C)	/	16.62–26.28	20.99–23.15	11.78–18.29
SSS (‰)	/	29.59–33.7	33.65–34.23	32.09–34.39
SBS (‰)	/	32.17–34.59	34.09–34.5	32–34.51
*Loliolus japonica*				
Depth (m)	29–83	67	33	15–16
SST (°C)	13.37–17.91	27.21	18.89	8.09–8.21
SBT (°C)	9.6–17.83	10.74	18.9	8.14–8.17
SSS (‰)	29.38–32.73	28.76	31.53	31.86–31.95
SBS (‰)	31.26–33.45	32.87	31.49	31.67–31.98

^a^ Abbreviations: SST, sea surface temperature; SSS, sea surface salinity; SBT, sea bottom temperature; SBS, sea bottom salinity.

**Table 3:** Seasonal data for catch per unit effort by weight (CPUE_w_; unit: g·h^−1^), number (CPUE_n_; unit: ind·h^−1^), and average individual weight (AIW; unit: g·ind^−1^) from autumn 2018 to summer 2019.

Measurement	Spring	Summer	Autumn	Winter
	*Uroteuthis duvauceli*			
Mean CPUE_w_ at collection stations	68.51	533.81	730.39	147.55
Value range of CPUE_w_	41.68–114.2	33–1752.54	38.8–3768.33	34.8–392
Mean CPUE_n_ at collection stations	3	8.67	40.38	3.5
Value range of CPUE_n_	1–6	3–19	2–370	1–8
Mean AIW	37.9	45.57	61.19	40.8
Value range of AIW	6.95–57.1	9.28–92.24	10.18–321.3	28–51.4
	*Uroteuthis edulis*			
Mean CPUE_w_ at collection stations	406.7	1059.07	737.33	162.93
Value range of CPUE_w_	12.3–7018.4	23.4–20,684.4	5.14–3742	17.5–747
Mean CPUE_n_ at collection stations	16.47	23.13	63.3	9.06
Value range of CPUE_n_	1–356	1–549	1–488	1–33
Mean AIW	50.04	67	71.87	22.74
Value range of AIW	6.26–123.1	5.6–175.41	0.8–384	2.95–59
	*Loliolus sumatrensis*			
Mean CPUE_w_ at collection stations	/	162.47	308.73	116.98
Value range of CPUE_w_	/	4.1–481	23.66–858	17.4–249.6
Mean CPUE_n_ at collection stations	/	13.14	22.8	8.83
Value range of CPUE_n_	/	1–47	2–60	1–36
Mean AIW	/	12.9	11.17	23.12
Value range of AIW	/	2–27	2.3–24.5	6.63–43.35
	*Loliolus japonica*			
Mean CPUE_w_ at collection stations	114.79	52	13.44	14.15
Value range of CPUE_w_	23.28–372.63	52	13.44	3.9–24.4
Mean CPUE_n_ at collection stations	4.75	2	5	1.5
Value range of CPUE_n_	2–13	2	5	1–2
Mean AIW	17.98	26	2.69	8.05
Value range of AIW	11.64–28.66	26	2.69	3.9–12.2

**Table 4:** Percentages of habitat loss, gain, and overall habitat (gain minus loss) for *Uroteuthis duvauceli*, *Uroteuthis edulis*, *Loliolus sumatrensis*, and *Loliolus japonica* under various climate scenarios (SSP126-2050, SSP126-2100, SSP245-2050, SSP245-2100, SSP370-2050, SSP370-2100, SSP585-2050, and SSP585-2100).

Scenario	*Uroteuthis duvauceli*			*Uroteuthis edulis*		
	Loss%	Gain%	Gain–Loss%	Loss%	Gain%	Gain–Loss%
SSP126-2050	−13.504	10.955	−2.549	0	26.674	26.674
SSP126-2100	−16.812	14.65	−2.162	−0.087	20.174	20.087
SSP245-2050	−13.705	12.101	−1.604	0	25.583	25.583
SSP245-2100	−25.09	20.564	−4.525	0	24.34	24.34
SSP370-2050	−10.683	12.072	1.389	−0.065	20.065	20
SSP370-2100	−61.378	2.764	−58.614	0	36.445	36.445
SSP585-2050	−16.855	15.208	−1.647	0	29.706	29.706
SSP585-2100	−55.521	1.575	−53.945	0	51.32	51.32
Scenario	*Loliolus sumatrensis*			*Loliolus japonica*		
	Loss%	Gain%	Gain–Loss%	Loss%	Gain%	Gain–Loss%
SSP126-2050	–0.491	71.333	70.842	–100	0	100
SSP126-2100	–0.697	111.88	111.183	–100	0	100
SSP245-2050	–0.62	76.705	76.085	–100	0	100
SSP245-2100	–5.656	193.233	187.577	–100	0	100
SSP370-2050	–2.04	76.756	74.716	–100	0	100
SSP370-2100	–14.618	270.713	256.095	–100	0	100
SSP585-2050	–0.517	90.393	89.876	–100	0	100
SSP585-2100	–25.413	274.897	249.483	–100	0	100

## Data Availability

The original contributions presented in this study are included in the article/supplementary material. Further inquiries can be directed to the corresponding author(s).

## Supplementary Materials

The following supporting information can be downloaded at https://www.mdpi.com/article/10.3390/ani15121744/s1: Figure S1. Calibration percentage (%) of TSS and ROC for *Uroteuthis duvauceli*. Figure S2. Calibration percentage (%) of TSS and ROC for *Uroteuthis edulis*. Figure S3. Calibration percentage (%) of TSS and ROC for *Loliolus japonica*. Figure S4. Calibration percentage (%) of TSS and ROC for *Loliolus sumatrensis*. Figure S5. Ratio values of TSS vs. ROC for *Uroteuthis duvauceli*. Figure S6. Ratio values of TSS vs. ROC for *Uroteuthis edulis*. Figure S7. Ratio values of TSS vs. ROC for *Loliolus japonica*. Figure S8. Ratio values of TSS vs. ROC for *Loliolus sumatrensis*. Figure S9. Box plots of the importance of environmental variables for *Uroteuthis duvauceli*. Figure S10. Box plots of the importance of environmental variables for *Uroteuthis edulis*. Figure S11. Box plots of the importance of environmental variables for *Loliolus japonica*. Figure S12. Box plots of the importance of environmental variables for *Loliolus sumatrensis*. Figure S13. The predicted habitat suitability of *Uroteuthis duvauceli* in different seasons. Figure S14. The predicted habitat suitability of *Uroteuthis edulis* in different seasons. Figure S15. The predicted habitat suitability of *Loliolus japonica* in different seasons. Figure S16. The predicted habitat suitability of *Loliolus sumatrensis* in different seasons. Figure S17. The predicted habitat suitability of *Uroteuthis duvauceli* in different climate scenarios. Figure S18. The predicted habitat suitability of *Uroteuthis edulis* in different climate scenarios. Figure S19. The predicted habitat suitability of *Loliolus japonica* in different climate scenarios. Figure S20. The predicted habitat suitability of *Loliolus sumatrensis* in different climate scenarios.
