# Supplementary material for: Seasonal Spatial Distribution Characteristics and Patterns of the Squid Uroteuthis duvauceli, Uroteuthis edulis, Loliolus sumatrensis, and Loliolus japonica in the Southern Yellow and East China Seas: Predictions Under Different Climate Scenarios
Source: Animals (Basel). 2025 Jun 13;15(12):1744. doi: 10.3390/ani15121744 (PMC12189684; doi:10.3390/ani15121744)
Supplement: Supplementary file 1 [file animals-15-01744-s001.zip › supplementary file S1.pdf]

Supplementary file S1

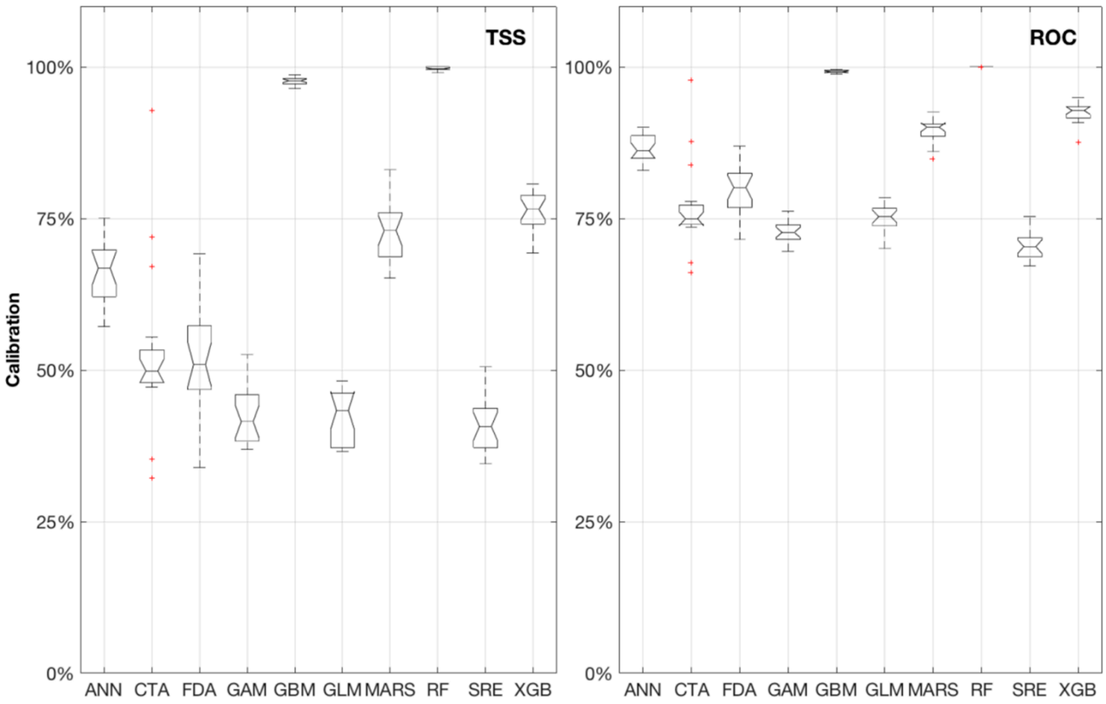

Figure S1. Calibration percentage (%) of TSS and ROC for *Uroteuthis duvauceli*.

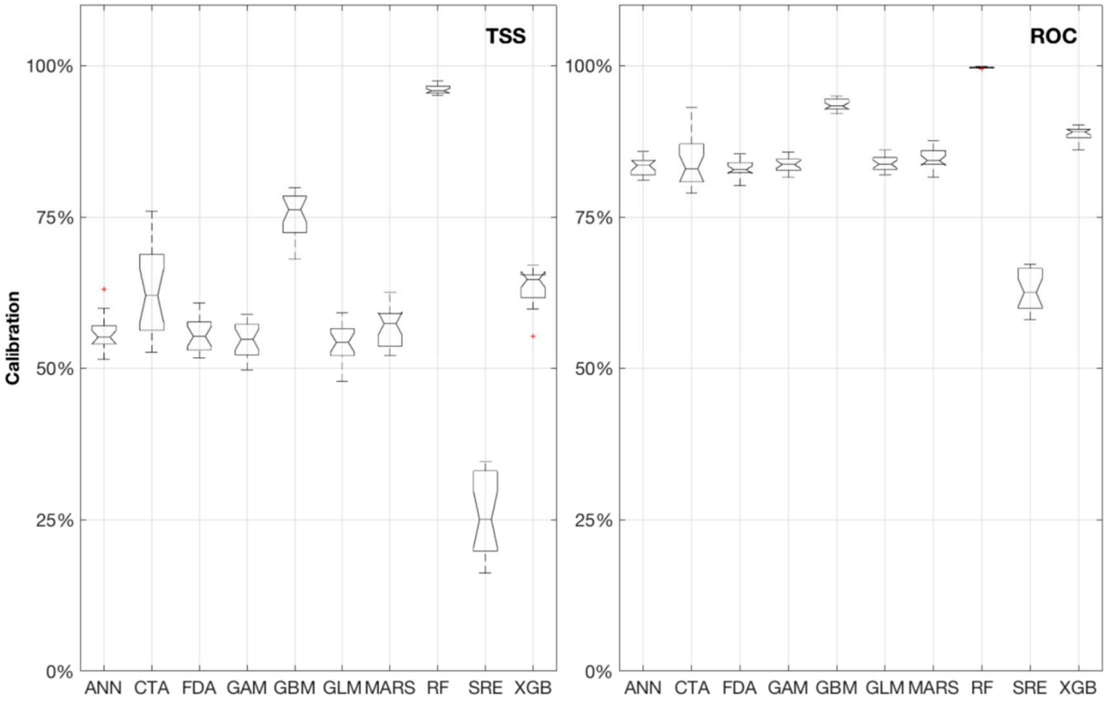

Figure S2. Calibration percentage (%) of TSS and ROC for the species *Uroteuthis edulis*.

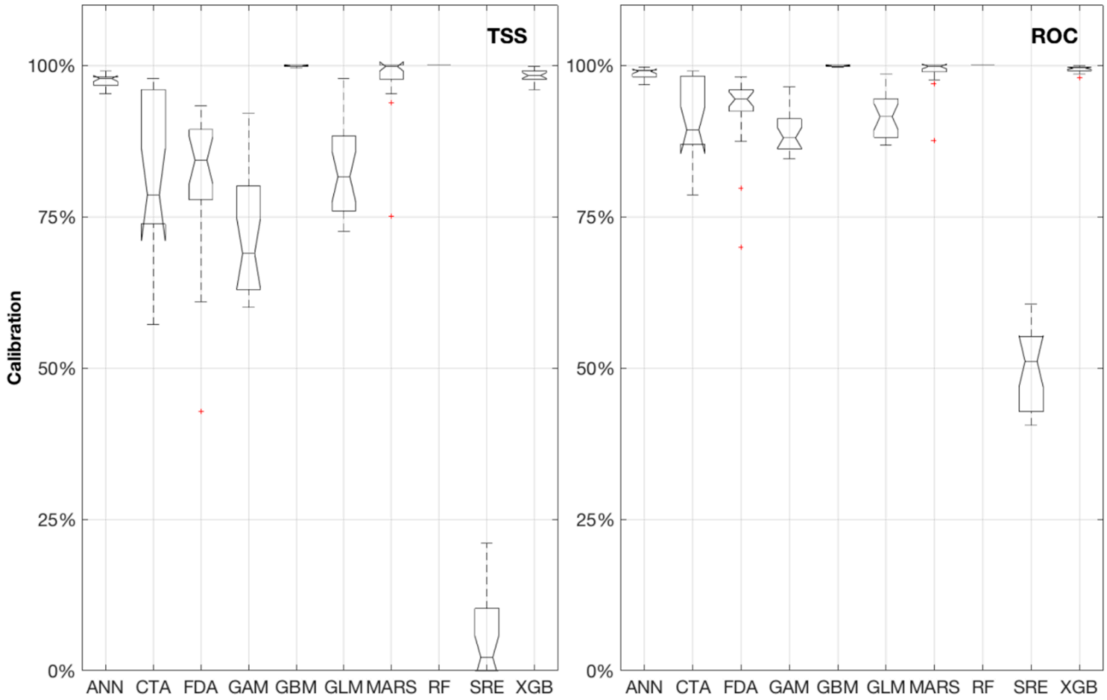

Figure S3. Calibration percentage (%) of TSS and ROC for the species *Loliolus japonica*.

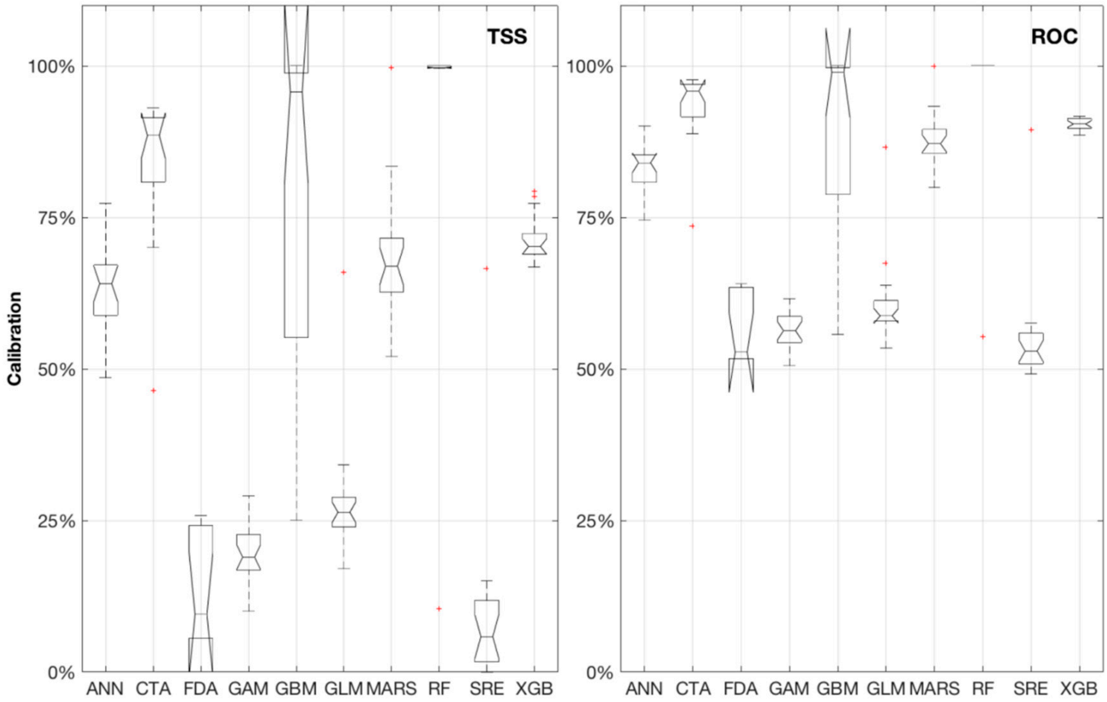

Figure S4. Calibration percentage (%) of TSS and ROC for the species *Loliolus sumatrensis*.

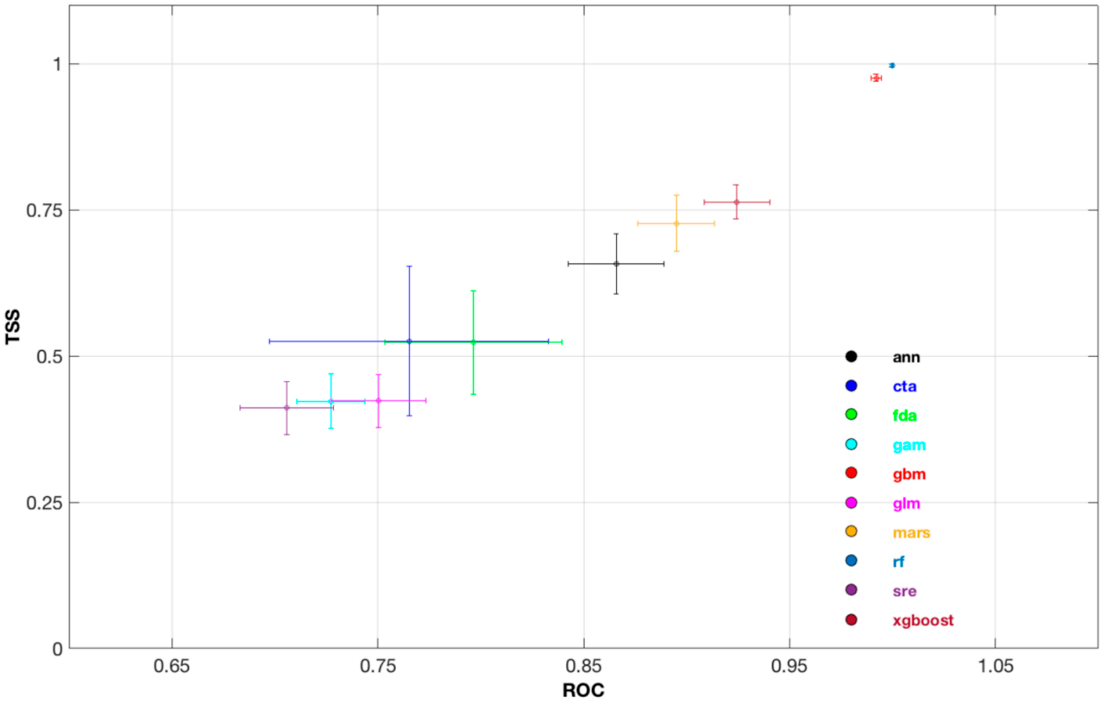

Figure S5. Ratio values of TSS vs. ROC for the species *Uroteuthis duvauceli*.

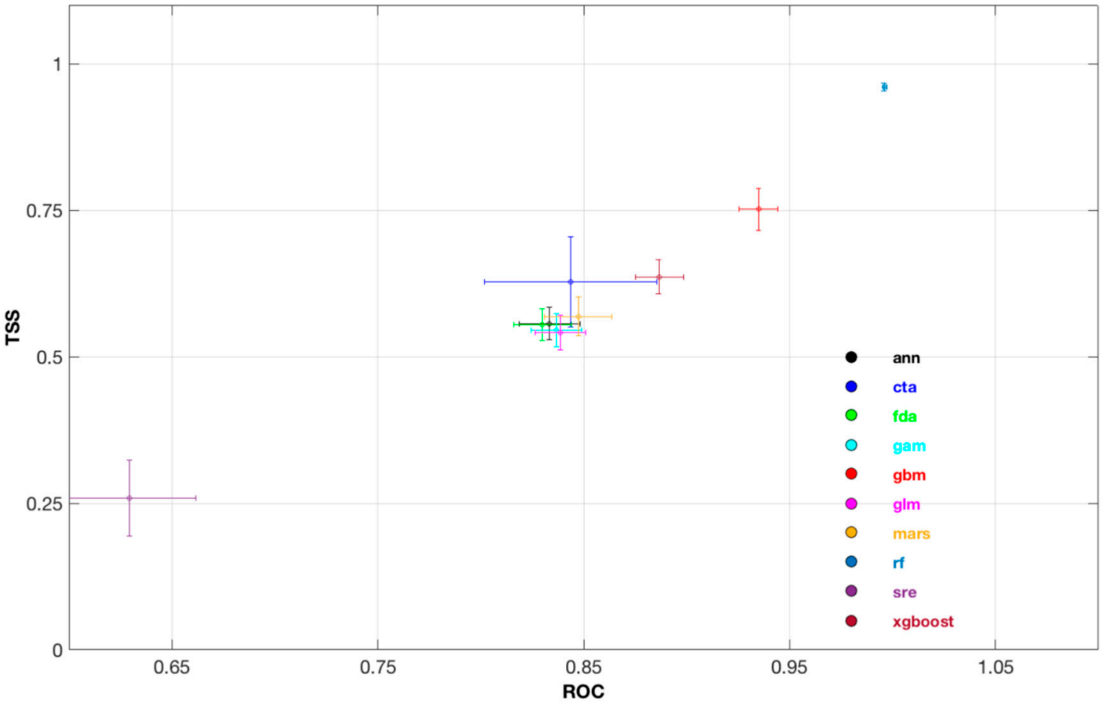

Figure S6. Ratio values of TSS vs. ROC for *Uroteuthis edulis*.

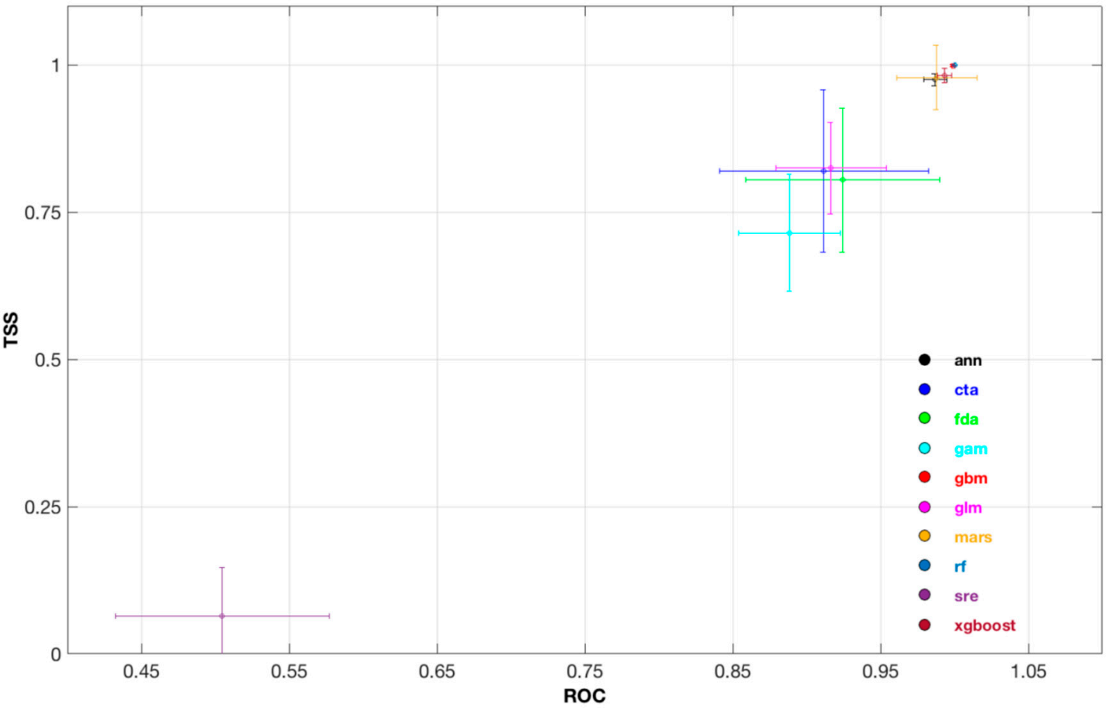

Figure S7. Ratio values of TSS vs. ROC for *Loliolus japonica*.

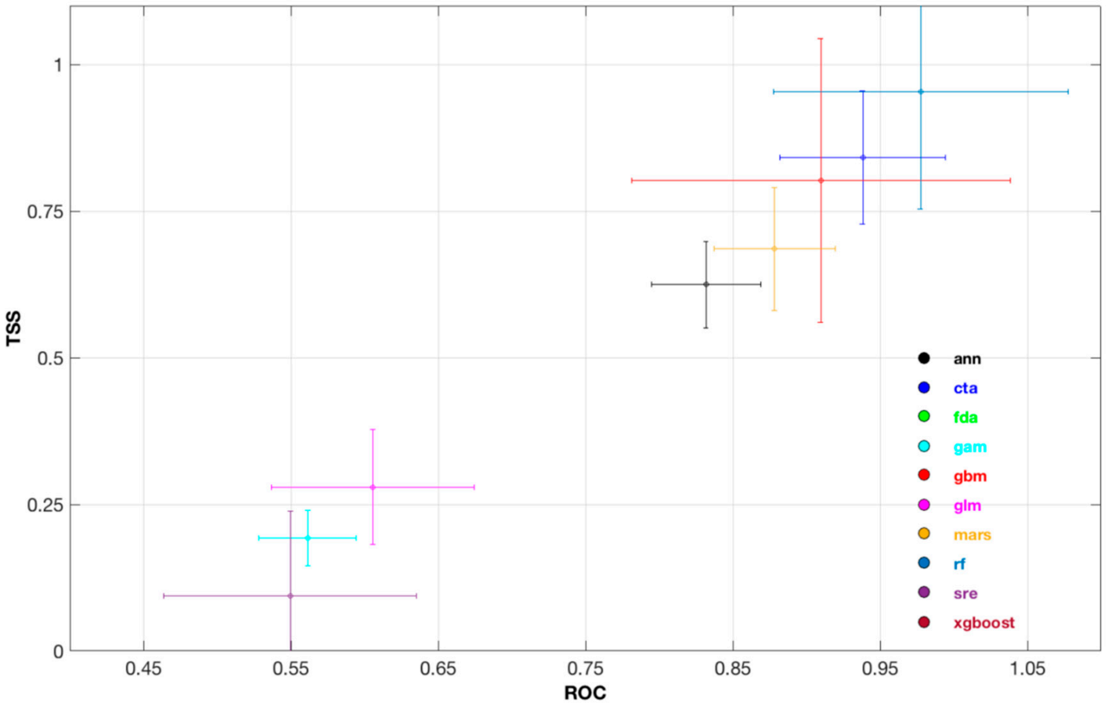

Figure S8. Ratio values of TSS vs. ROC for *Loliolus sumatrensis*.

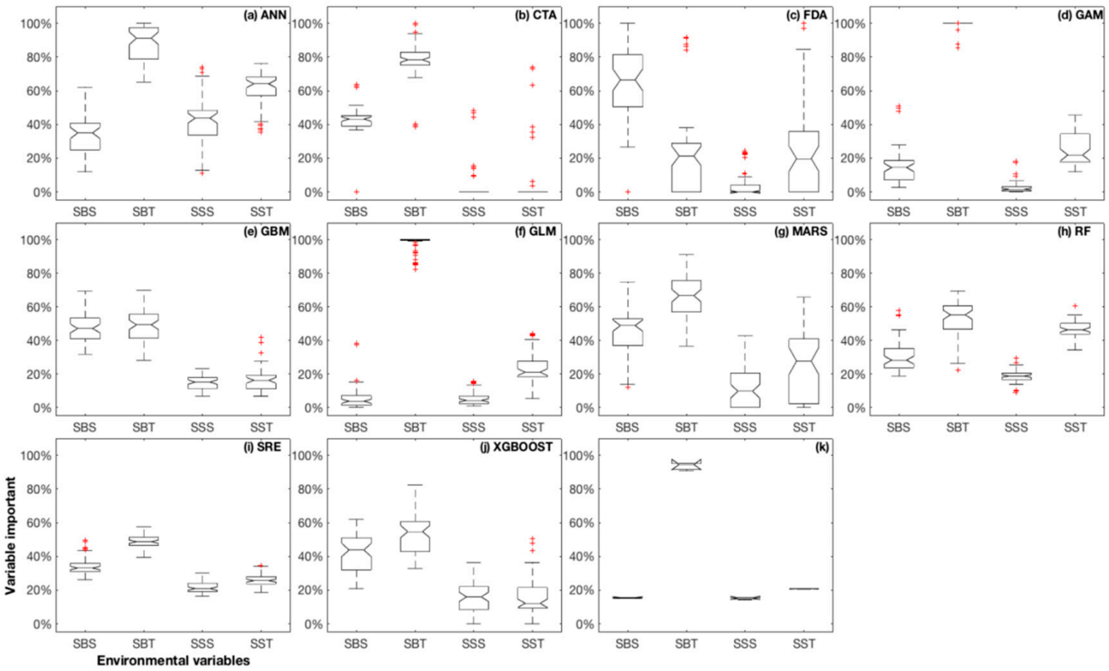

Figure S9. Box plots of the importance of environmental variables for the species *Uroteuthis duvauceli*.

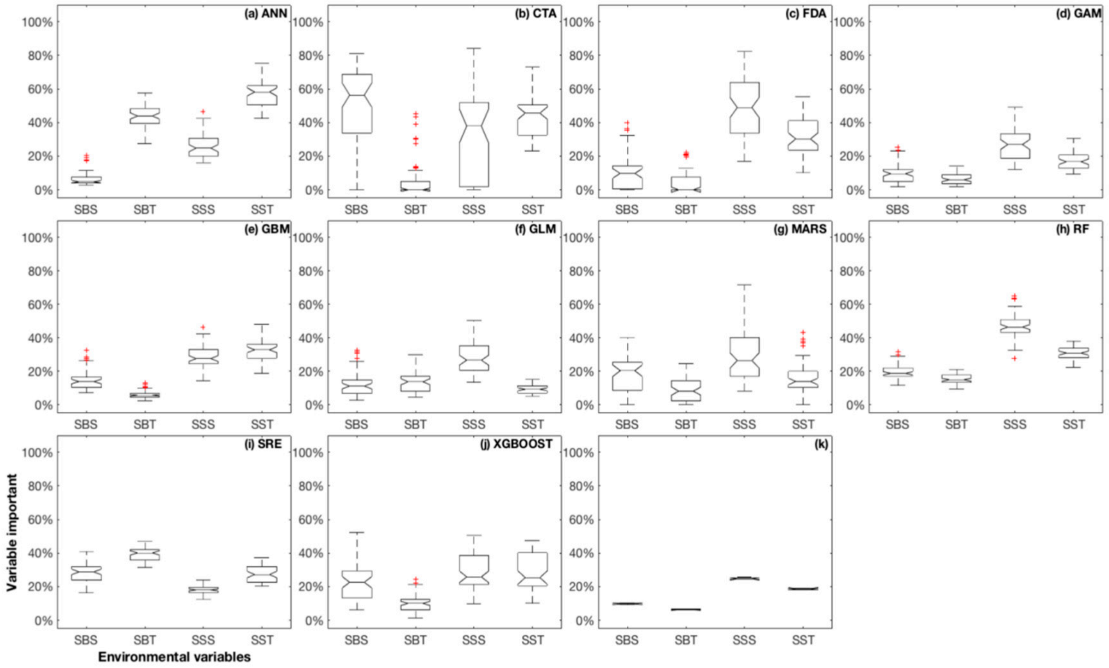

Figure S10. Box plots of the importance of environmental variables for *Uroteuthis edulis*.

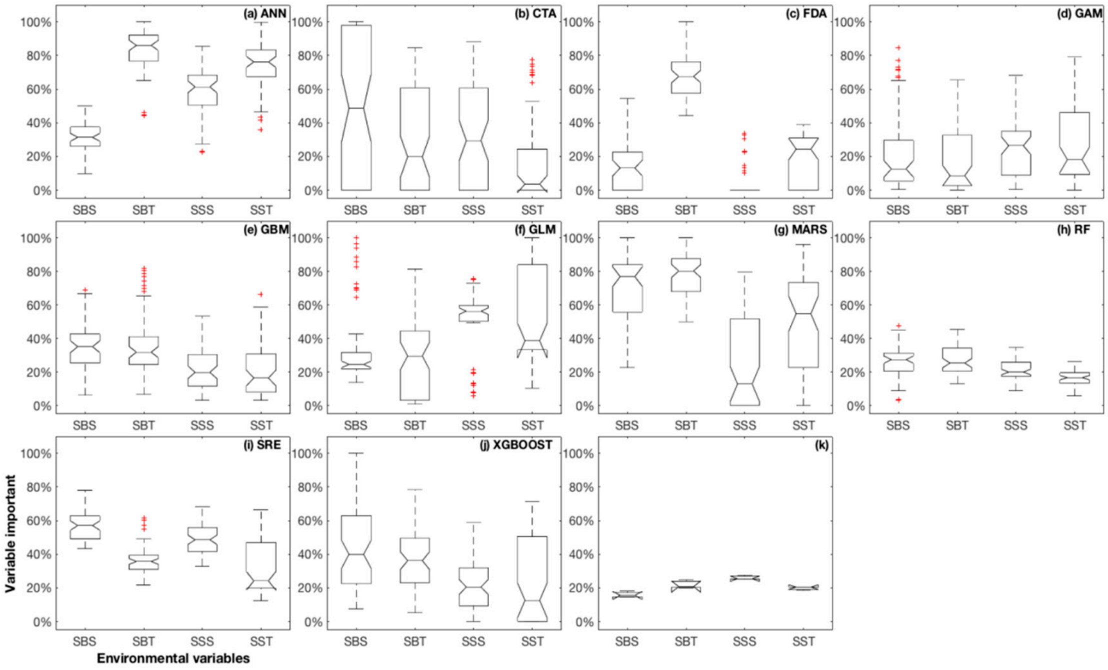

Figure S11. Box plots of the importance of environmental variables for *Loliolus japonica*.

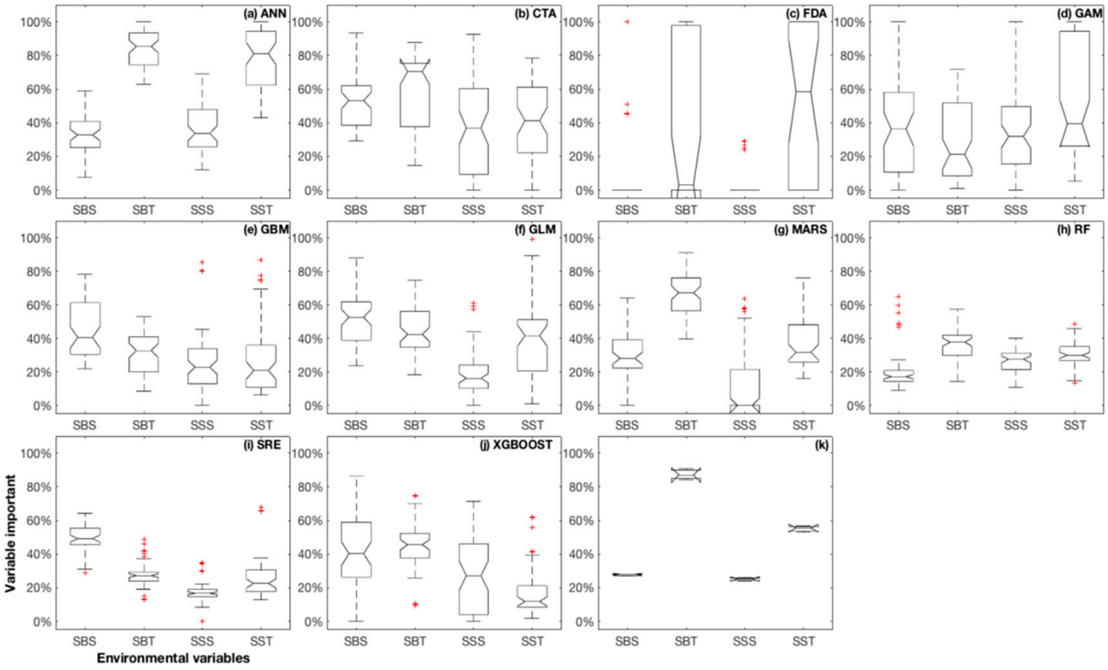

Figure S12. Box plots of the importance of environmental variables for *Loliolus sumatrensis*.

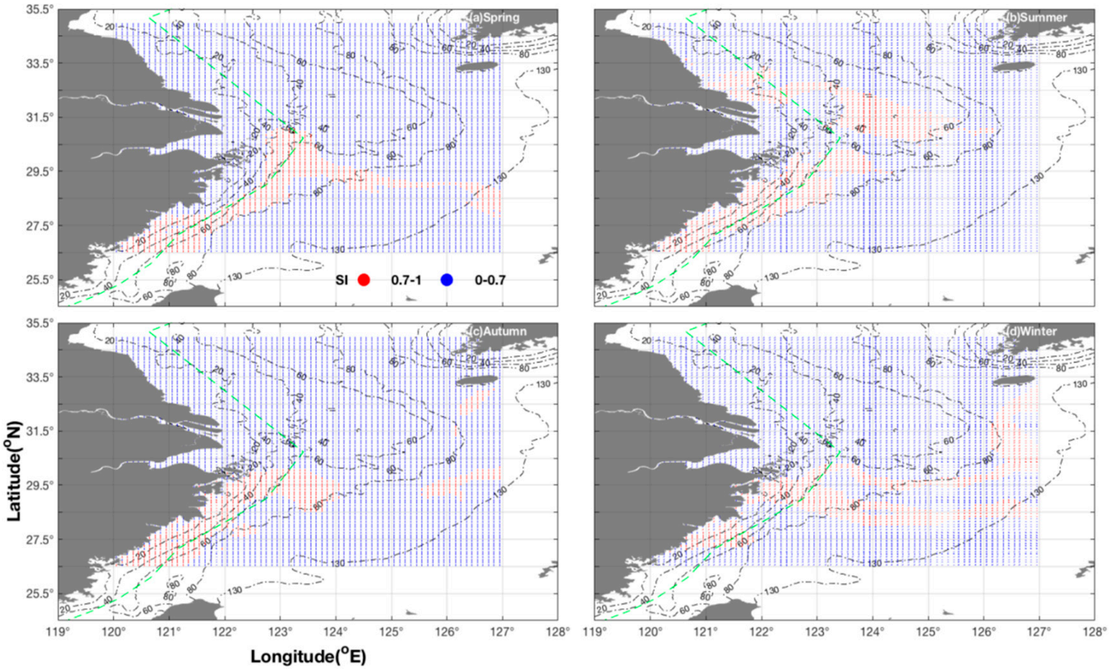

Figure S13. The predicted habitat suitability of *Uroteuthis duvauceli* in different seasons.

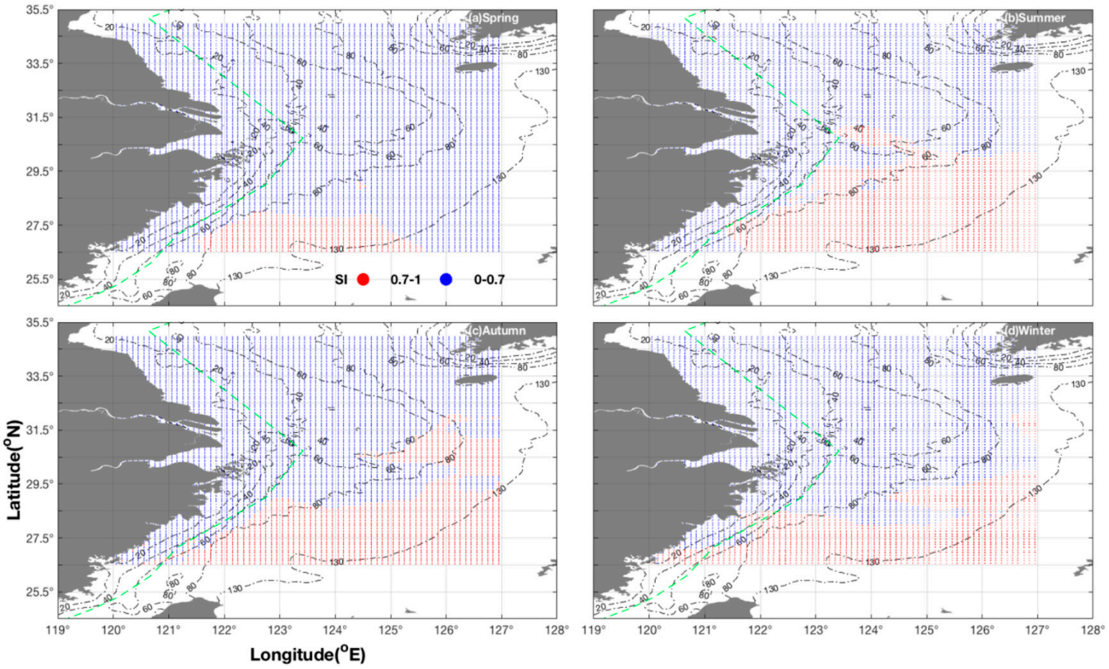

Figure S14. The predicted habitat suitability of *Uroteuthis edulis* in different seasons.

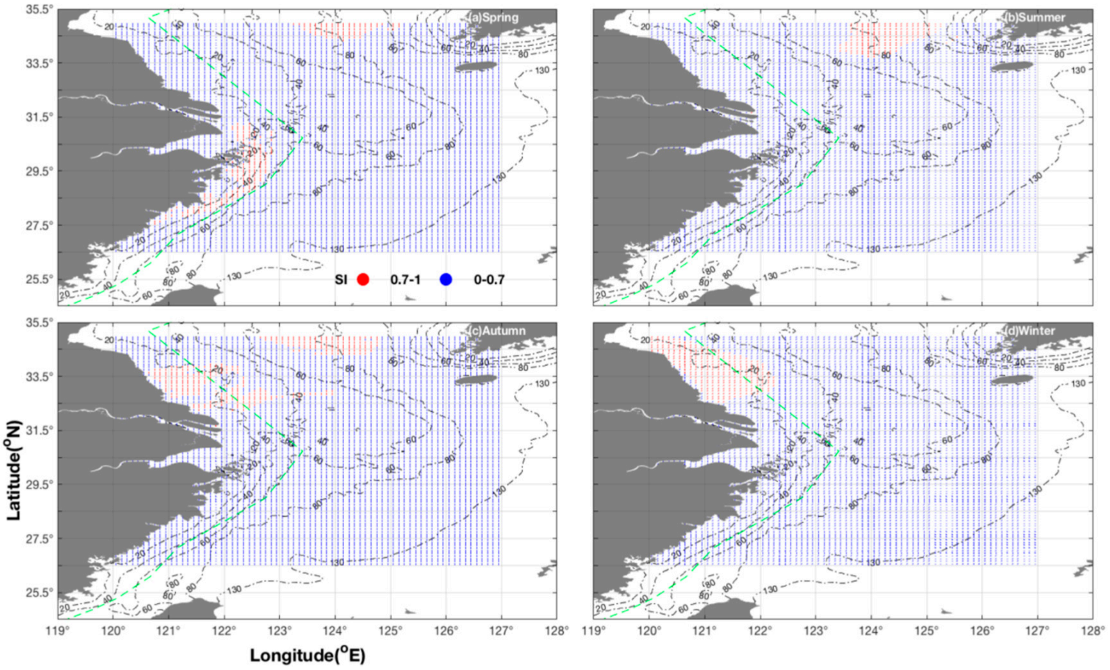

Figure S15. The predicted habitat suitability of *Loliolus japonica* in different seasons.

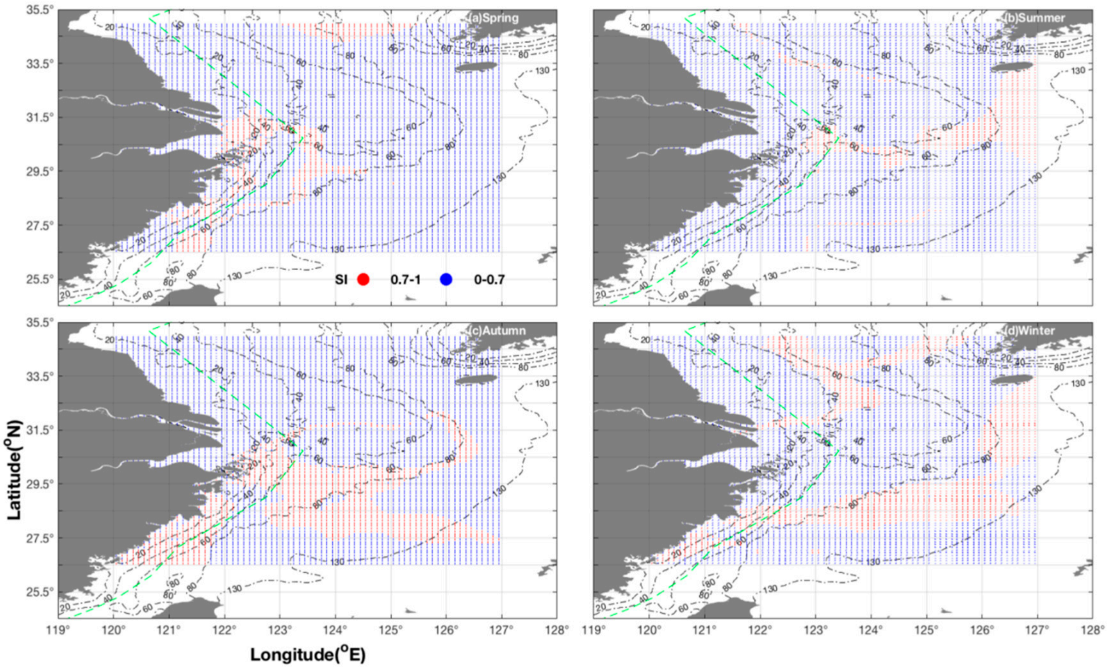

Figure S16. The predicted habitat suitability of *Loliolus sumatrensis* in different seasons.

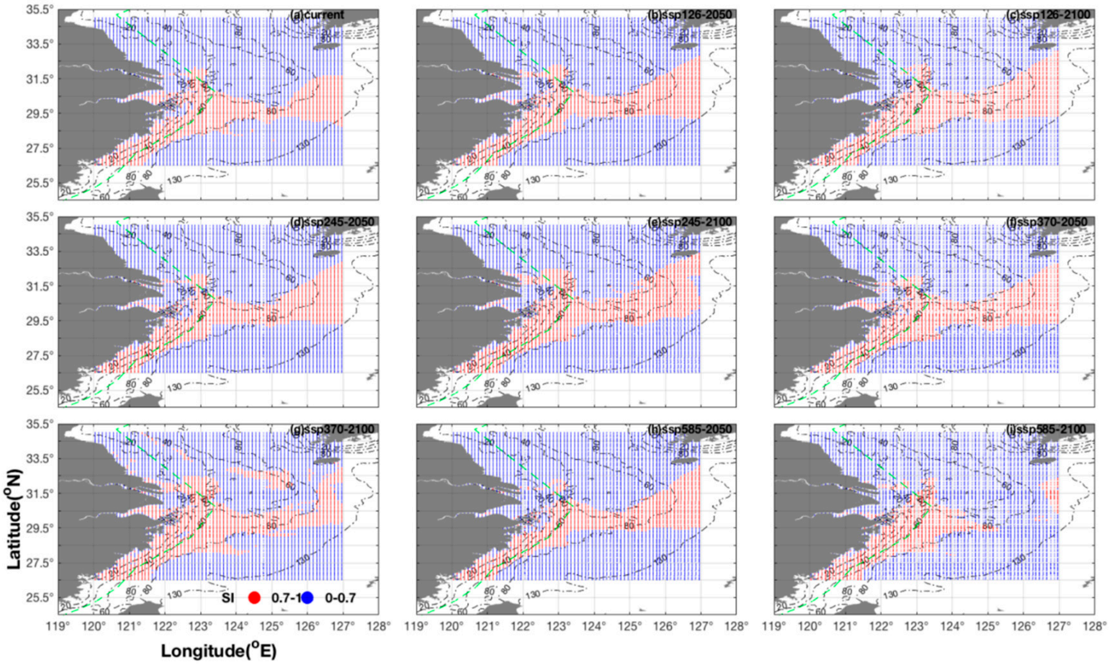

Figure S17. The predicted habitat suitability of *Uroteuthis duvauceli* in different climate scenarios.

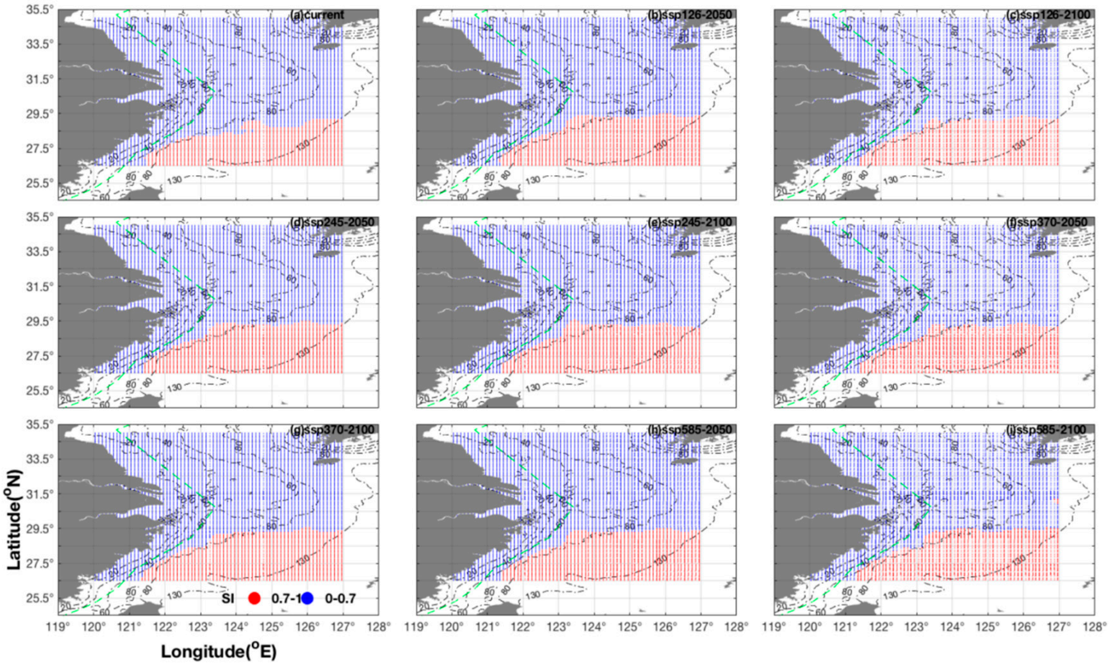

Figure S18. The predicted habitat suitability of *Uroteuthis edulis* in different climate scenarios.

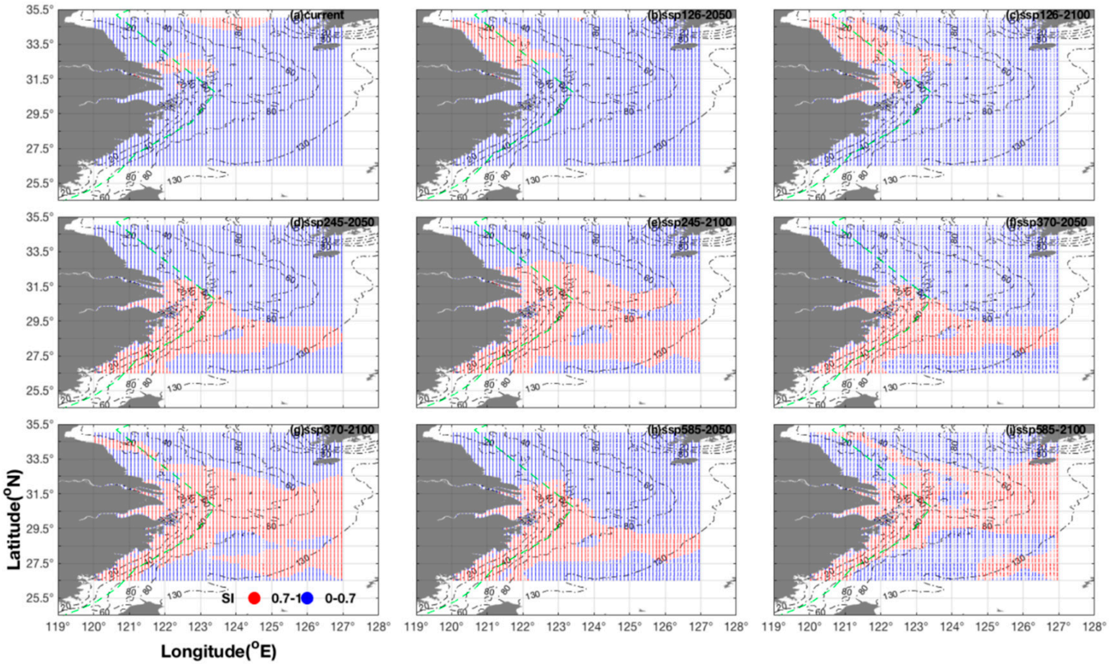

Figure S19. The predicted habitat suitability of *Loliolus japonica* in different climate scenarios.

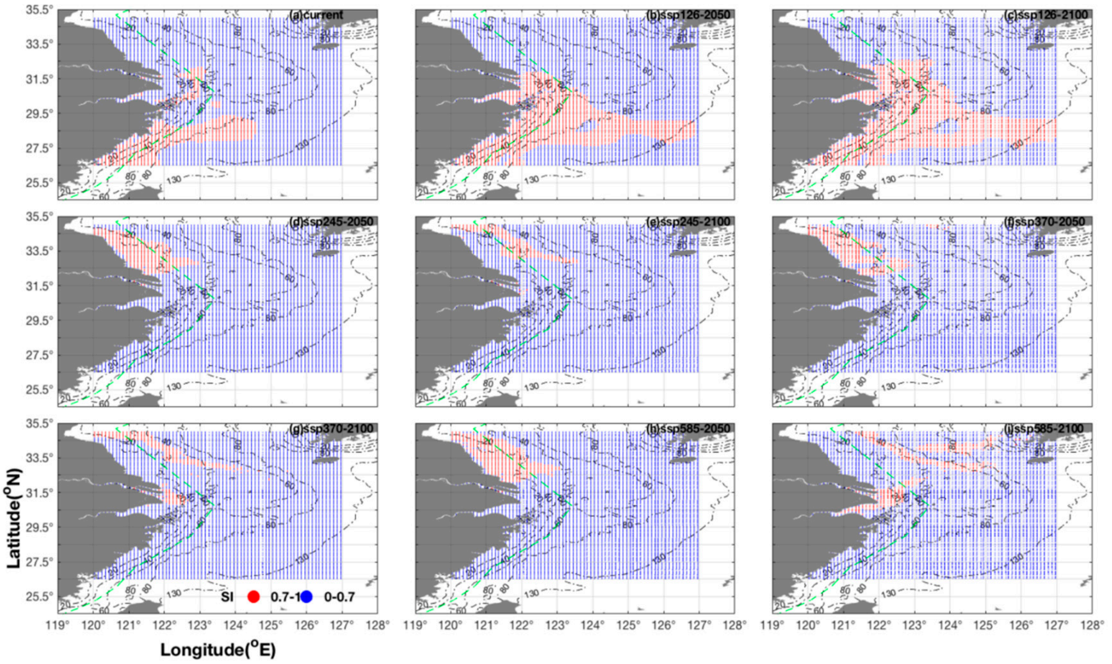

Figure S20. The predicted habitat suitability of *Loliolus sumatrensis* in different climate scenarios.
